# Supplementary material for: Diversity and complexity of arthropod references in haiku
Source: PLoS One. 2024 Apr 3;19(4):e0298865. doi: 10.1371/journal.pone.0298865 (PMC10990216; doi:10.1371/journal.pone.0298865)
Supplement: S1 Table — Columns represent (L to R): taxon, including common names; number of described species in each taxon [78, 79]; percent of total relevant arthropod species; number of occurrences for each taxon in iNaturalist [22]; percent of relevant arthropod occurrences in iNaturalist; number of references in the 2500 haiku corpus for each taxon; percent of all arthropod references in the corpus. Rows in bold had zero references in haiku. Rows highlighted in yellow are taxa with few species and no references in haiku. Rows in orange represent taxa that are surprisingly underrepresented. (PDF) [file pone.0298865.s001.pdf]

Table S 1: **Arthropod representation in haiku**

Taxon (common names)	# spp.	% spp.	# iNat	% iNat	# references	% references
Arachnida (spiders, scorpions, ticks)	112,201	9.697%	920,265	6.623%	220	10.49%
**Archaeognatha (bristletails)**	**513**	**0.044%**	**546**	**0.004%**	**0**	**0.00%**
Blattodea (cockroaches, termites)	7,314	0.632%	38,217	0.275%	38	1.56%
Coleoptera (beetles, fireflies, scarabs)	387,100	33.454%	1,578,768	11.363%	311	12.75%
Collembola (springtails)	8,130	0.703%	10,044	0.072%	2	0.08%
Dermaptera (earwigs)	1,978	0.171%	17,530	0.126%	5	0.20%
Diplura (diplurans)	800	0.069%	26	<0.001%	1	0.04%
Diptera (mosquitoes, midges, flies)	159,294	13.767%	644,823	4.641%	314	12.87%
**Embioptera (webspinners)**	**464**	**0.040%**	**381**	**0.003%**	**0**	**0.00%**
Ephemeroptera (mayflies, shadflies)	3,240	0.280%	4,510	0.032%	11	0.45%
Hemiptera (aphids, scale, cicadas, bugs)	103,590	8.952%	980,879	7.060%	192	7.87%
Hymenoptera (sawflies, wasps, bees, ants)	116,861	10.099%	1,387,881	9.989%	347	14.22%
Isopoda: Oniscidea (woodlice, pill bugs)	3,710	0.321%	87233	0.628%	4	0.16%
Lepidoptera (moths, butterflies, caterpillars)	157,424	13.605%	6,244,880	44.945%	510	20.90%
Mantodea (mantises, mantids)	2,400	0.207%	82,546	0.594%	36	1.48%
Mecoptera (scorpionflies, hangingflies)	757	0.065%	6,830	0.049%	1	0.04%
**Megaloptera (dobsonflies, hellgrammites)**	**354**	**0.031%**	**15,349**	**0.110%**	**0**	**0.00%**
Myriapoda (millipedes, centipedes)	11,885	1.027%	66,992	0.482%	20	0.82%
Neuroptera (lacewings)	5,868	0.507%	37,592	0.271%	5	0.20%
**Notoptera (ice crawlers, heelwalkers)**	**55**	**0.005%**	**28**	**<0.001%**	**0**	**0.00%**
Odonata (dragonflies, damselflies, darners)	5,899	0.510%	1,302,595	9.375%	135	5.53%
Orthoptera (crickets, katydids, grasshoppers)	24,276	2.098%	424,464	3.055%	204	8.36%
Phasmatodea (stick insects)	3,029	0.262%	18,140	0.131%	5	0.20%
**Plecoptera (stoneflies)**	**3,788**	**0.327%**	**1,058**	**0.008%**	**0**	**0.00%**
Protura (coneheads)	804	0.069%	1	<0.001%	1	0.04%
Psocodea (bark lice, parasitic lice)	10,822	0.935%	5,640	0.041%	12	0.49%
**Raphidioptera (snakeflies)**	**254**	**0.022%**	**694**	**0.005%**	**0**	**0.00%**
Siphonaptera (fleas)	2,075	0.179%	73	0.001%	25	1.02%
**Strepsiptera (twisted-wing parasites)**	**609**	**0.053%**	**131**	**0.001%**	**0**	**0.00%**
Thysanoptera (thrips)	6,019	0.520%	503	0.004%	1	0.04%
**Trichoptera (caddisflies)**	**14,999**	**1.296%**	**9,015**	**0.065%**	**0**	**0.00%**
**Zoraptera (angel insects)**	**37**	**0.003%**	**5**	**<0.001%**	**0**	**0.00%**
Zygentoma (silverfish, firebrats)	561	0.048%	6,755	0.049%	4	0.16%
total =	1,157,110	100%	13,894,394	100%	2,440	100.00%

Columns represent (L to R): taxon, including common names; number of described species in each taxon [1, 2]; percent of total relevant arthropod species; number of occurrences for each taxon in iNaturalist [3]; percent of relevant arthropod occurrences in iNaturalist; number of references in the 2500 haiku corpus for each taxon; percent of all arthropod references in the corpus. Rows in **bold** had zero references in haiku. Rows highlighted in yellow are taxa with few species and no references in haiku. Rows in orange represent taxa that are surprisingly underrepresented.

References

[1] Zhang, Zhi-Qiang (2011) Phylum Arthropoda von Siebold, 1848 In: Zhang, Z.-Q.(Ed.) Animal biodiversity: An outline of higher-level classification and survey of taxonomic richness. Zootaxa 3148(1): 99–103. DOI: `https://doi.org/10.11646/zootaxa.3148.1.14`

[2] Spyros Sfendourakis and Stefano Taiti (2015) Patterns of taxonomic diversity among terrestrial isopods. ZooKeys 515: 13–25. DOI: `https://doi.org/10.3897/zookeys.515.9332`

[3] iNaturalist contributors and iNaturalist (2022) iNaturalist Research-grade Observations. iNaturalist.org Occurrence dataset. Available at `https://doi.org/10.15468/ab3s5x` Accessed via GBIF.org on 2023-01-04.
