# Supplementary figures and images for: Diversity and complexity of arthropod references in haiku
Source: PLoS One. 2024 Apr 3;19(4):e0298865. doi: 10.1371/journal.pone.0298865 (PMC10990216; doi:10.1371/journal.pone.0298865)

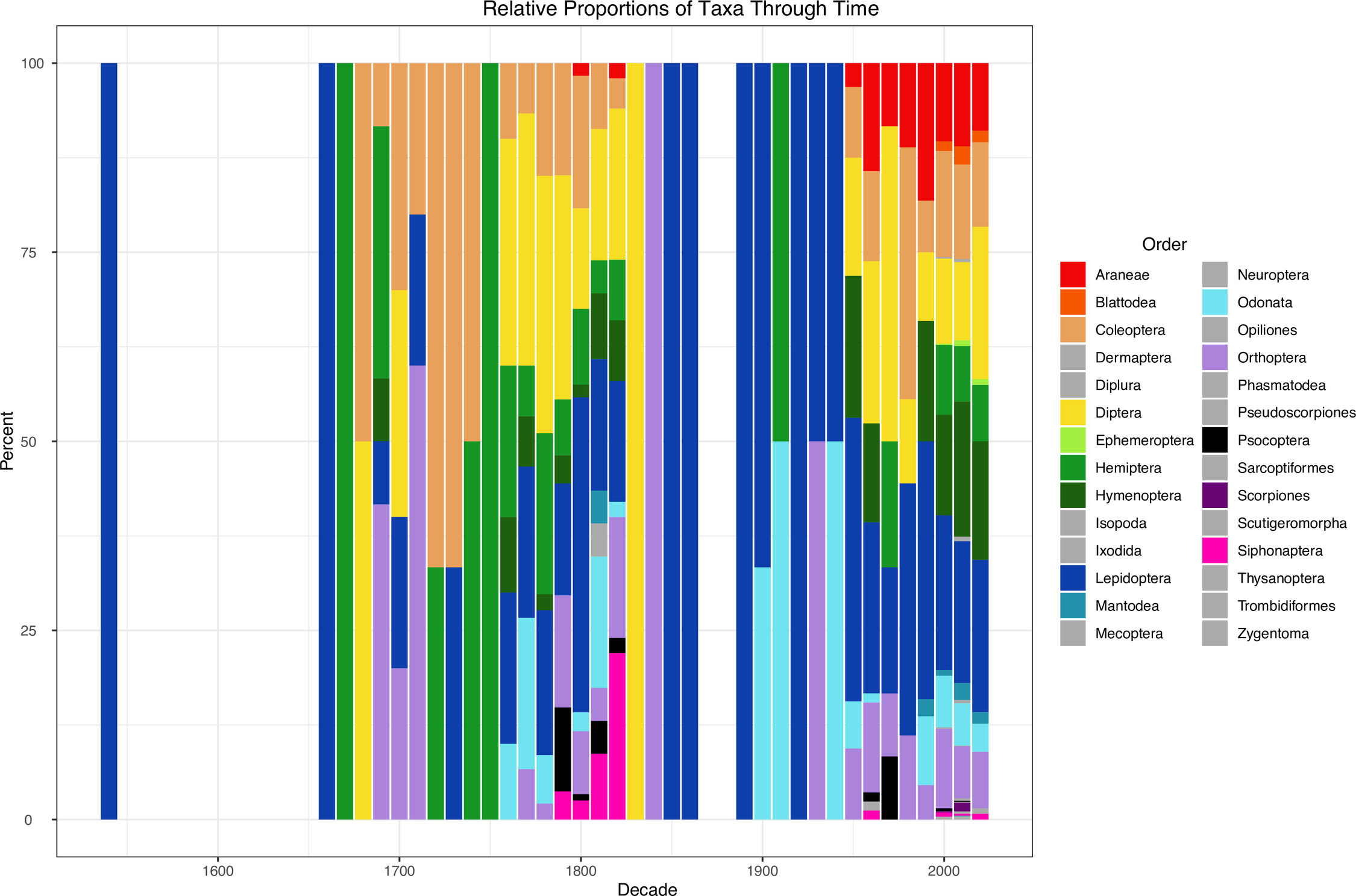

Supplement: S1 Fig — This figure illustrates the proportion of haiku that reference any particular taxon at a particular period of time. (TIF) [file pone.0298865.s004.tif]

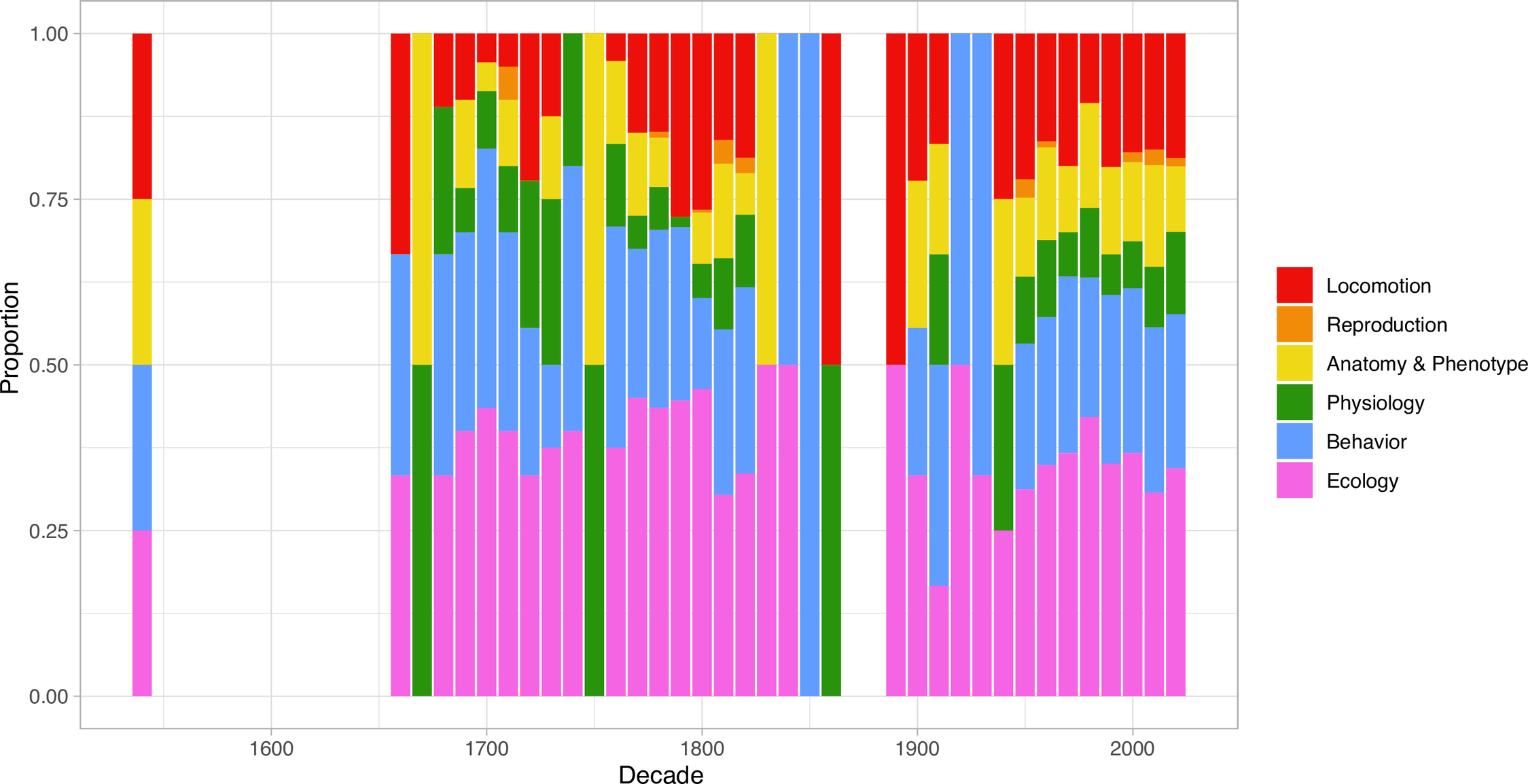

Supplement: S2 Fig — This figure illustrates the proportion of haiku that reference the major trait categories in each decade. (TIF) [file pone.0298865.s005.tif]

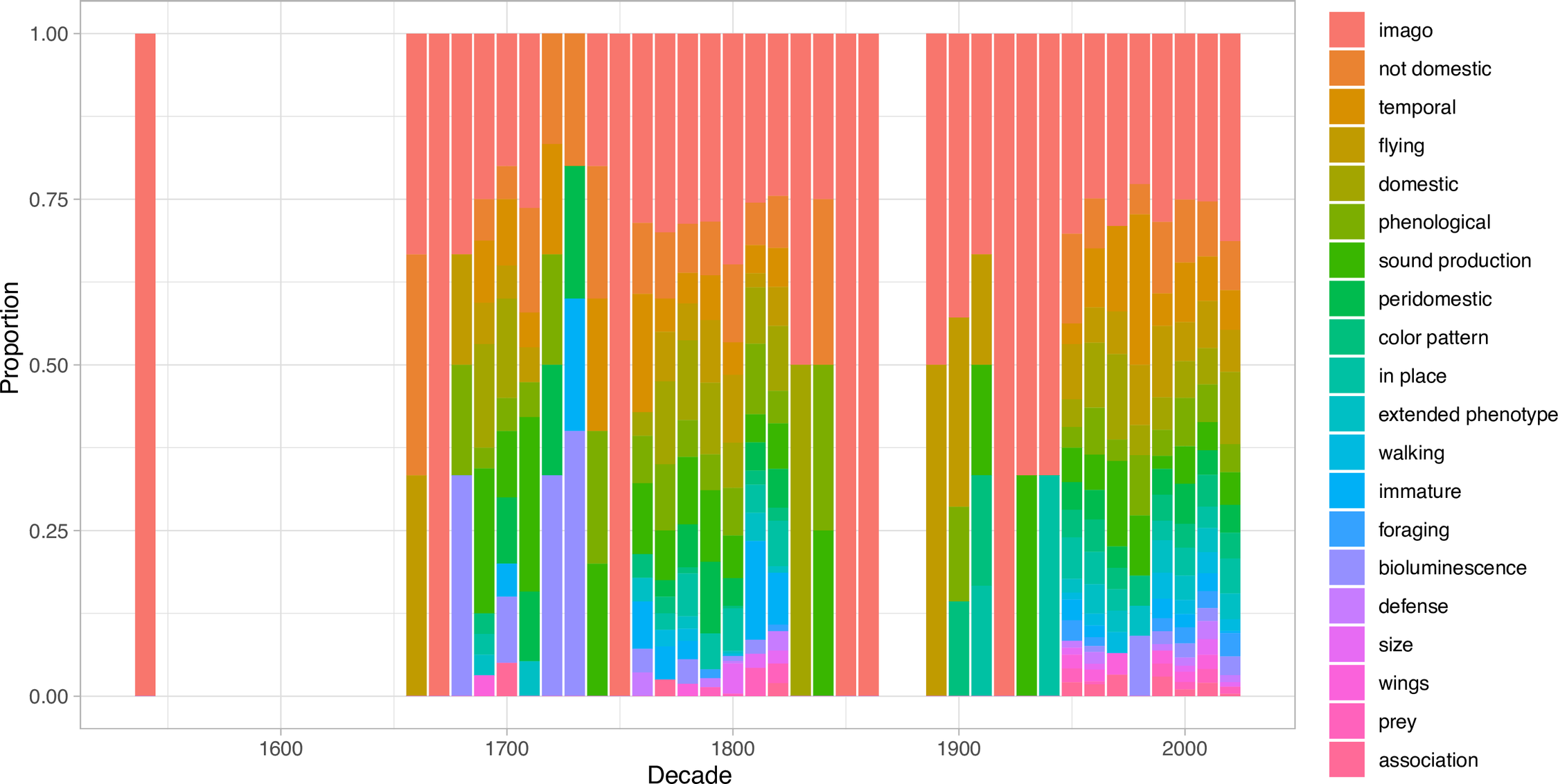

Supplement: S3 Fig — This figure illustrates the proportion of haiku that reference the top 20 traits in each decade. (TIF) [file pone.0298865.s006.tif]

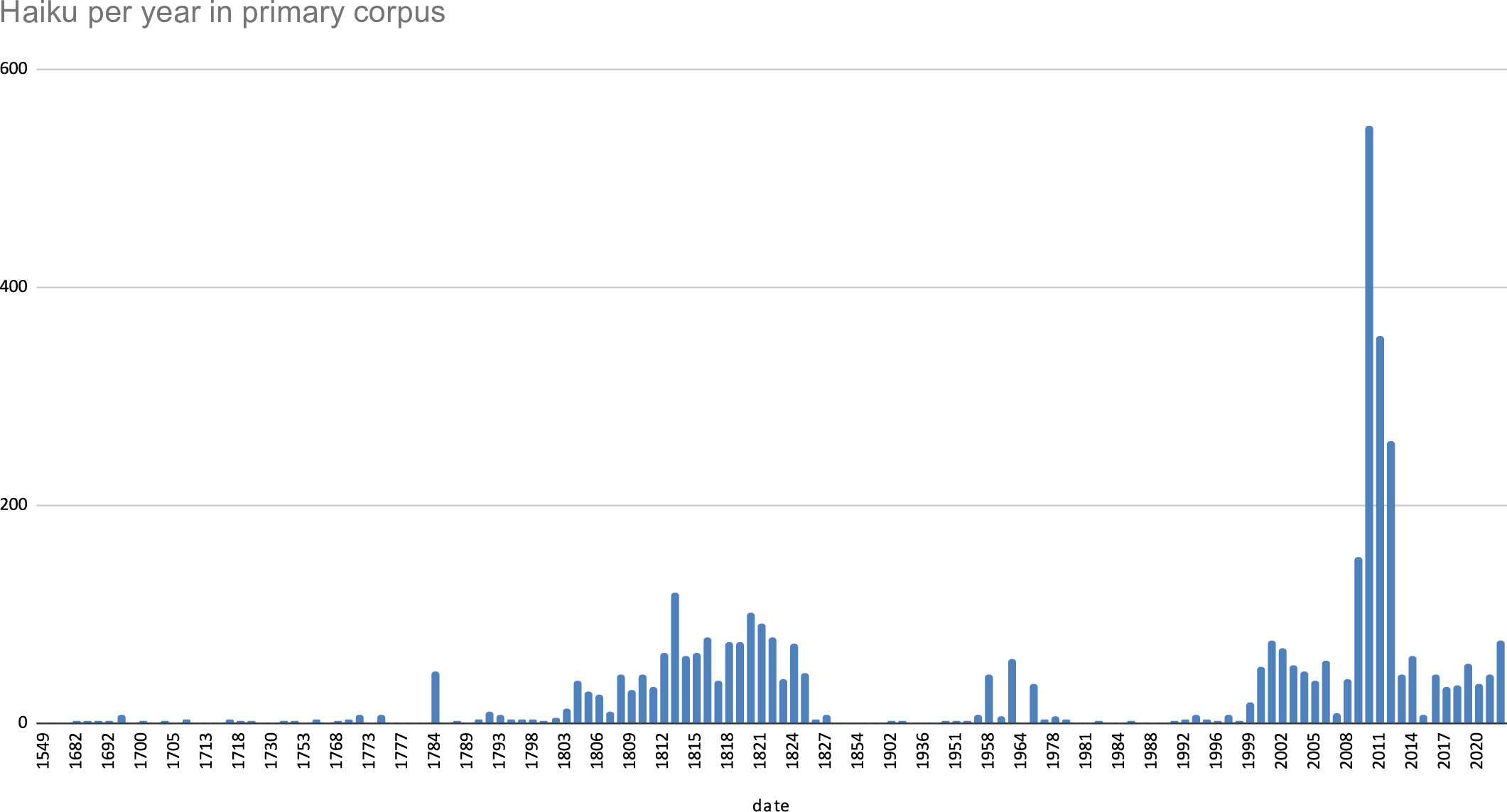

Supplement: S4 Fig — This histogram illustrates the number of poems per year that are represented in the primary corpus. (TIF) [file pone.0298865.s007.tif]
