# Supplementary material for: Diversity and complexity of arthropod references in haiku
Source: PLoS One. 2024 Apr 3;19(4):e0298865. doi: 10.1371/journal.pone.0298865 (PMC10990216; doi:10.1371/journal.pone.0298865)
Supplement: S1 Appendix — This document describes the strategies and resources used in constructing the corpora of haiku that reference arthropods. This document is also available through Penn State’s institutional repository, ScholarSphere: https://doi.org/10.26207/gpxg-q347. (PDF) [file pone.0298865.s008.pdf]

Preamble

This document describes the strategies and resources used in constructing a corpus of haiku that reference arthropods. The subsequent corpus was analyzed by Andrew R. Deans and Laura Porturas in 2022.

Database organization

Our sources for haiku are described below. Relevant results from these sources were parsed into several fields in a spreadsheet (.csv), using Dublin Core metadata terms (https://www.dublincore.org/specifications/dublin-core/dcmi-terms/) as headers, where possible:

1. *creator* - name of the poet
2. *source* - bibliographic information, with URI or DOI if available
3. *date* - year of publication
4. *poem* - the text of the poem itself, pasted without regard to formatting
5. *contributor* - the person responsible for adding the poem to the corpus
6. *notes* - any comments made by the contributor

Sources

haikuguy.com

David G. Lanoue (Xavier University) is an authority on haiku and has assembled what is probably the world's largest database of haiku—12,020 total haiku, on 2 November 2022—by Kobayashi Issa: http://haikuguy.com/issa/. Among this collection are hundreds of poems that reference insects.

The database was queried using the keywords listed below. The search results were scanned for relevant poems, which were added to a spreadsheet. Poems listed as "year unknown" were excluded from the data set.

Keywords used:
ant; aphid; arachnid; backswimmer; bee; beetle; birdwing; biter; blue; borer; bristletail; bug; butterfl; buzz; caddis; caterpillar; centipede; chafer; chelicerae; chrysalis; cicada; cide; cobweb; coccinella; cocoon; collembolan; conehead; cricket; curculio; cutworm; daddy; damselfly; darner; dauber; dipluran; dragonfly; drone; Drosophila; dun; dustywing; earwig; eggar; exoskeleton; feelers; firebrat; firefl; flea; fly; gall; gnat; grub; hairstreak; harvestman; hawker; hellgrammite; hive; hopper; hornet; horntail; insect; japyx; jewelwing; katydid; ked; lacewing; lady; larva; lerp; lice; locust; longhorn; longicorn; looper; louse; maggot; mantis; mayfly; midge; millipede; miner; mite; mosquito; moth; naiad; no-see-um; oarsman; orb; owlet; pest; prominent; proturan; pupa; pseudoscorpion; punkie; roach; rolypoly; sawfly; sawyer; scarab; scorpion; silkworm; silverfish; skimmer; skipper; skwala; sow; spider; spinneret; spreadwing; springtail; sting; stonefly; strider; stylops; swallowtail; swarm; tarantula; termite; thrips; tick; trig; underwing; venom; walking stick; wasp; web; weevil; worm; yellowjacket

terebess.hu

Terebess Asia Online (TAO) provides multiple translations of Bashō, Buson, and other haiku poets. Citation:

- Terebess, Gábor (2021) Terebess Asia Online (TAO). Available at: https://web.archive.org/web/20210615221645/https://terebess.hu/english/haiku/haiku.html Accessed 8 April 2022

Translations available through TAO were gathered from the following resources, which were also consulted on an *ad hoc* basis:

- Blyth, Reginald Horace (1964a) *A History of Haiku: Volume One: From the Beginnings up to Issa*. The Hokuseido Press
- Blyth, Reginald Horace (1964b) *A History of Haiku: Volume Two: From Issa up to the Present*. The Hokuseido Press
- Ginsberg, Allen (1978) Mostly Sitting Haiku. 1st ed. (Xtras, no. 6) Paterson, New Jersey: From Here Press. 23 pages. ISBN 0-89120-010-X
- Hakutani, Yoshinobu (1996) *Richard Wright and Racial Discourse*. University of Missouri Press, Columbia. 312 pages. ISBN-13: 978-0826210593
- Hoffmann, Y. (1986) Japanese death poems. Rutland, Vt: C.E. Tuttle Co. ISBN-13: 978-0804831796
- Merwin, W. S.; Lento, Takako; Buson, Yosa (2013) *Collected Haiku of Yosa Buson*. Copper Canyon Press, Port Townsend, Washington. xviii, 247 pages. ISBN-13: 978-1556594267
- Ogburn, F. (1998) Richard Wright's unpublished haiku: A world elsewhere. *MELUS*, 23(3), 57–81. https://doi.org/10.2307/467678
- Persinger, Allan (2013) Foxfire: the Selected Poems of Yosa Buson, a Translation. Theses and Dissertations. 748. https://dc.uwm.edu/etd/748
- Saito, Takafumi; Nelson, William R. (2006) *1020 Haiku in Translation: The Heart of Basho, Buson and Issa*. ISBN-13: 978-1419627651
- Sawa, Yuki ; Marcombe Shiffert, Edith (2007) *Haiku Master Buson*. Published by White Pine Press, Buffalo, NY. 178 pages. ISBN-13: 978-0893460129
- Stryk, Lucien (1993) *Cage of Fireflies: Modern Japanese Haiku*. Greece: Swallow Press. 118pages. ISBN-13: 978-0804009775
- Ueda, Makoto (1998) *The Path of Flowering Thorn: The Life and Poetry of Yosa Buson*. Stanford: Stanford University Press. 226 pages. ISBN-13: 978-0804730426

The relevant documents were downloaded and manually searched for references to the arthropod keywords listed above.

Haiku Foundation Digital Library

The Haiku Foundation (https://thehaikufoundation.org/) hosts many digital publications that include a diversity of haiku in English.

Available resources were browsed manually for relevant haiku, which were copied or transcribed into a spreadsheet. Some "haiku" were actually extracted, if they could stand alone as haiku, from haibun or renku. The following resources were accessed in April 2022:

- Aoyagi, Fay (2003) "Chrysanthemum Love," The Haiku Foundation Digital Library, accessed April 13, 2022, https://thehaikufoundation.org/omeka/items/show/5
- Anakiev, Dimitar (2002) "At The Tombstone," *The Haiku Foundation Digital Library*, accessed April 13, 2022, https://thehaikufoundation.org/omeka/items/show/4
- Ashraf, Hifsa and Summers, Alan (2019) "The Comfort of Crows," The Haiku Foundation Digital Library, accessed April 14, 2022, https://thehaikufoundation.org/omeka/items/show/6247
- Barton, Jeb (1997) "Short Distance, Long Journey," *The Haiku Foundation Digital Library*, accessed April 13, 2022, https://thehaikufoundation.org/omeka/items/show/7
- Bell, Amanda (2016) "Undercurrents by Amanda Bell," *The Haiku Foundation Digital Library*, accessed April 13, 2022, https://thehaikufoundation.org/omeka/items/show/6412
- Bernath, Jimi (2012) "face of the snail," The Haiku Foundation Digital Library, accessed April 13, 2022, https://thehaikufoundation.org/omeka/items/show/6379
- Blyth, R. H., "A History of Haiku Volume One," The Haiku Foundation Digital Library, accessed April 13, 2022, https://thehaikufoundation.org/omeka/items/show/215
- Bostok, Janice (2002) "Amongst the Graffiti: Collected Haiku and Senryu
- 1972--2002," *The Haiku Foundation Digital Library*, accessed April 13, 2022, https://thehaikufoundation.org/omeka/items/show/12
- Burleigh, David (2013) "James Kirkup (1918–2009)," *The Haiku Foundation Digital Library*, accessed April 13, 2022, https://thehaikufoundation.org/omeka/items/show/14
- Chambers, Paul (2021) "The Dry Bones," *The Haiku Foundation Digital Library*, accessed April 13, 2022, https://thehaikufoundation.org/omeka/items/show/6399
- Brendon Kent, Michael Smeer Layout: Marina Bellini, Michael Smeer, eds. (2021) "Spirits Up! selected haiku, senryu & haiga," The Haiku Foundation Digital Library, accessed April 13, 2022, https://thehaikufoundation.org/omeka/items/show/6299
- France, B. A. and PréJean, Orrin (2021) "Retweets," The Haiku Foundation Digital Library, accessed April 13, 2022, https://thehaikufoundation.org/omeka/items/show/6290
- Hackett, James W. (1958) "6/11/58," The Haiku Foundation Digital Library, accessed April 14, 2022, https://thehaikufoundation.org/omeka/items/show/6271
- Hackett, James W. (1958) "Haiku 1958," The Haiku Foundation Digital Library, accessed April 14, 2022, https://thehaikufoundation.org/omeka/items/show/6270
- Hackett, James W. (1968) "Bug Haiku," The Haiku Foundation Digital Library, accessed August 10, 2022, https://thehaikufoundation.org/omeka/items/show/133 [not added to corpus due to partial overlap with other Hackett references]
- Kocher, Philomene (2021) "new boards on the barn," The Haiku Foundation Digital Library, accessed April 14, 2022, https://thehaikufoundation.org/omeka/items/show/6278

- Kocher, Philomene and Fraticelli, Marco (2021) “Changing Demographics,” The Haiku Foundation Digital Library, accessed April 14, 2022, https://thehaikufoundation.org/omeka/items/show/6219
- Kray, Lavan (2020) “Respiro,” The Haiku Foundation Digital Library, accessed April 14, 2022, https://thehaikufoundation.org/omeka/items/show/6217
- Pirie, Pearl (2020) “Not Quite Dawn,” The Haiku Foundation Digital Library, accessed April 14, 2022, https://thehaikufoundation.org/omeka/items/show/6208
- Pizzarelli, Alan (1975) “Zenryu,” The Haiku Foundation Digital Library, accessed April 14, 2022, https://thehaikufoundation.org/omeka/items/show/6275
- Pizzarelli, Alan (1989) “the flea circus,” The Haiku Foundation Digital Library, accessed April 14, 2022, https://thehaikufoundation.org/omeka/items/show/6274
- Savina, Zoe (2022) “A History of Greek Haiku,” The Haiku Foundation Digital Library, accessed April 15, 2022, https://thehaikufoundation.org/omeka/items/show/1398
- Simser, Guy (2015) “War is the Father of us All,” The Haiku Foundation Digital Library, accessed April 14, 2022, https://thehaikufoundation.org/omeka/items/show/6225
- Singh, Ram Krishna, “Lantern in the Sky,” *The Haiku Foundation Digital Library*, accessed April 13, 2022, https://thehaikufoundation.org/omeka/items/show/6417
- Singh, Ram Krishna (2021) “A Lone Sparrow,” The Haiku Foundation Digital Library, accessed April 14, 2022, https://thehaikufoundation.org/omeka/items/show/6203
- Spring Street Haiku Group (2010) “Suspiciously Small,” *The Haiku Foundation Digital Library*, accessed April 13, 2022, https://thehaikufoundation.org/omeka/items/show/6397
- Spring Street Haiku Group (2017) “a tiny wobble,” The Haiku Foundation Digital Library, accessed April 13, 2022, https://thehaikufoundation.org/omeka/items/show/6390
- Spring Street Haiku Group (2019) “the silence of the cymbals,” *The Haiku Foundation Digital Library*, accessed April 13, 2022, https://thehaikufoundation.org/omeka/items/show/6389
- Spring Street Haiku Group (2019) “the weight of moon light,” *The Haiku Foundation Digital Library*, accessed April 13, 2022, https://thehaikufoundation.org/omeka/items/show/6388
- Sugita, Naho (2021) “Goldfish's Sigh,” The Haiku Foundation Digital Library, accessed April 14, 2022, https://thehaikufoundation.org/omeka/items/show/6198
- Tartamella, Pietro, ed. (2022) “A History of Haiku in Italy,” The Haiku Foundation Digital Library, accessed April 15, 2022, https://thehaikufoundation.org/omeka/items/show/1637
- tripi, vincent (2001) “monk & i,” The Haiku Foundation Digital Library, accessed April 13, 2022, https://thehaikufoundation.org/omeka/items/show/6285
- Wirth, Klaus-Dieter (2020) “Stimmen der Steine/Voices of Stones/Voix de pierres/Voces de piedras: 145 Haiku by Klaus-Dieter Wirth,” The Haiku Foundation Digital Library, accessed April 13, 2022, https://thehaikufoundation.org/omeka/items/show/6288
- Young, Kristine (1979) “Through a Window,” The Haiku Foundation Digital Library, accessed April 15, 2022, https://thehaikufoundation.org/omeka/items/show/173
- Yovu, Peter (2004) “Turn to the Earth,” The Haiku Foundation Digital Library, accessed April 15, 2022, https://thehaikufoundation.org/omeka/items/show/113

- ZabRatyński`, Rafał and Kania, Robert (2022) “A History of Haiku in Poland,” The Haiku Foundation Digital Library, accessed April 15, 2022, https://thehaikufoundation.org/omeka/items/show/1697
- Zheng, J Q (2008–2010) “Haiku Page,” The Haiku Foundation Digital Library, accessed April 15, 2022, https://thehaikufoundation.org/omeka/items/show/5989
- Zheng, J Q (2006) “Found Haiku (from Eudora Welty’s Delta Wedding),” The Haiku Foundation Digital Library, accessed April 15, 2022, https://thehaikufoundation.org/omeka/items/show/1539
- Zubrinic, Darko (2022) “Haiku Poetry in Croatia,” The Haiku Foundation Digital Library, accessed April 15, 2022, https://thehaikufoundation.org/omeka/items/show/790

Haiku Society of America

The Haiku Society of America (HSA; https://www.hsa-haiku.org/) lists numerous haiku from past competitions, and back issues of their haiku journal, *Frogpond*, are accessible. These two resources yielded many poems that reference terrestrial arthropods.

- https://www.hsa-haiku.org/frogpond/previousissues.html
- https://www.hsa-haiku.org/haikuawards/henderson.htm
- https://www.hsa-haiku.org/senryuawards/senryu.htm
- https://www.hsa-haiku.org/frogpond/museumawardscollection.html (volume 31.2 through volume 35.1)

The website was browsed manually, to find haiku that reference terrestrial arthropods.

The Heron’s Nest

The Heron’s Nest (https://www.theheronsnest.com/) is a freely accessible online haiku journal, with issues going back to 1999. Issues were browsed manually for relevant haiku. Volumes I–VIII were browsed manually for relevant haiku, up through Vol. VIII issue 2.

Cattails

Cattails (http://www.cattailsjournal.com/archives.html) is a freely accessible online haiku journal, with issues going back to 2014. Issues from its first year were browsed manually for relevant haiku.

failed haiku

failed haiku is a journal of English senryu. Volumes 72, 75–77, and 80 were browsed manually for relevant haiku. Available at: https://failedhaiku.com/failed-haiku-issues-past-and-present/. Accessed 17 August 2022

Echidna Tracks

Echidna Tracks (https://echidnatracks.com/) is a freely accessible online haiku journal in Australia, with issues going back to 2018. Issues 1–6 were browsed manually for relevant haiku.

The Mamba

The Mamba (https://africahaikunetwork.wordpress.com/contact/) is a freely accessible online haiku journal in Africa, with issues going back to 2016. Issues 1–8 were browsed manually for relevant haiku.

Shamrock (Irish Haiku Society'sJournal)

Shamrock (https://shamrockhaiku.webs.com/archive.htm) is a freely accessible online haiku journal for the Irish Haiku Society, with issues going back to 2007. Not added to the corpus for this study.

Richard Wright

Richard Wright wrote thousands of haiku in the years before his death, in 1960. More than 800 of these haiku were published posthumously, in 1998:

- Wright, R., Hakutani, Y., & Tener, R. L. (1998). *Haiku: This other world*. New York: Arcade Pub. ISBN-13: 978-1-55970-445-8

Haiku from this publication were read aloud and transcribed using the dictation feature in macOS 12.2.1 (Apple, Inc., Cupertino, CA USA). Transcribed poems were edited as necessary on the fly and entered into a spreadsheet.

Millikin University Haiku

Dr. Randy Brooks teaches courses on haiku at Millikin University and hosts a website with poems from these students. The provenance of each poem is well-documented, and the poets comprise a diverse group of people. The website was browsed manually, to find haiku that reference terrestrial arthropods.

- Brooks, R. et al. (2022) Millikin University Haiku. Available at: https://web.archive.org/web/20211222154504/http://brooksbookshaiku.com/MillikinHaiku/index.html Accessed 12 April 2022.

Hexapod Haiku

Every year from 2009–2012 and 2019–present, Andrew R. Deans, with help from colleagues, has organized a haiku contest that focuses on arthropod-themed poems. The poems come from poets around the world and of many ages. Poems sorted largely into two categories: 12 and under, 13 and older. Entries from 2009–2012 were included in the corpus.

References for future studies

- Hearn, L. (1901) A Japanese miscellany. [see chapter on dragonflies; lots of haiku] https://digital.cincinnatilibrary.org/digital/collection/p16998coll8/id/751
