# Supplementary material for: Diversity and complexity of arthropod references in haiku
Source: PLoS One. 2024 Apr 3;19(4):e0298865. doi: 10.1371/journal.pone.0298865 (PMC10990216; doi:10.1371/journal.pone.0298865)
Supplement: S2 Appendix — This document describes the process used to score haiku for biological complexity and taxon. This document is also available through Penn State’s institutional repository, ScholarSphere: https://doi.org/10.26207/04h1-a695. (PDF) [file pone.0298865.s009.pdf]

Preamble

This document describes the process used by Andrew R. Deans and Laura Porturas to score haiku for biological complexity and taxon.

Identifier

Each poem is assigned an identifier, consisting of an abridgement of the poet's name (creator) + year poem created (date) + arthropod term. The purpose of the identifier is to give each poem a unique reference string. For example:

> evening cherry blossoms--
> the ants also
> have a place to sleep
>
> by Kobayashi Issa (1811)

The poem above would get this identifier: `Issa1811Ants`. If more than one poem was written about ants in 1811 by Issa, each identifier would be appended by a sequential number: `Issa1811Ants1`, `Issa1811Ants2`, etc.

If the date of the poem is unknown, the final year of the poet's life is used as the date.

Taxon

The lowest level taxon for each poem was determined based on diagnostic words. In this haiku by Issa, for example:

> temple fly mimic--
> hands
> praying beads

The word "fly" and the phrase "mimic hands praying" together suggest some type of short-horned fly (Brachycera), possibly a muscoid (Muscoidea). It could confidently be scored as `Brachycera`.

For this haiku, however:

> gentle summer wind
> sunlight warms tiny bodies
> the beetles scurry

The information is insufficient to determine the taxon below `Coleoptera`. The diagnostic words and the lowest level taxon were recorded for each poem.

Biological complexity

Each of these would be scored as yes or no, depending on whether this aspect of the arthropod's biology was mentioned, alluded to, or was likely at least partly understood by the poet when the haiku was written.

class	trait	description	examples from poems
locomotion<br><br>n/8	LOCOMOTION	some unspecified locomotion referenced (if more specific, then score this as yes and select appropriate trait(s) below)	*such is life--<br>the burning field's bugs<br>a feast for birds*
	ambulatory	walking, crawling	*sunfree circumambulation<br>of the sink*
	cursory	running	*their stuttering quickness*
	saltatory	jumping	*Over the gravestone –<br>a grasshopper is jumping*
	natatory	swimming	*… in a rainwater* barrel<br>*mosquito larvae*
	skating	skating on water surface	*gliding on top<br>of* mountain *water* too...<br>*water strider*
	flight	flying	*… black butterfly flitting*
	in place	resting, guarding, etc.	*… cicada on a branch*
reproduction<br><br>n/6	REPRODUCTION	Some aspect of reproductive biology mentioned (score as yes if any other reproduction traits are present)	*the silver-spotted skipper<br>searches for first mate*
	courtship	nuptial flight, nuptial gifts, males recruiting females	*termite season<br>my dog laps up<br>waterborne wings*
	mating	in copula, e.g.	*Like jeweled beads for our eyes<br>Hatch, fly, eat, mate, lay*

	oviposition	laying eggs	*… lay your eggs come August, Little Tiger Moth*
	hatching		*... push out the purse … that contains a hundred jellylike nymphs*
	brood care		*…as all concerned mothers of young spiders will do*
anatomy / phenotype<br><br>n/11	ANATOMY / PHENOTYPE	some unspecified aspect of the arthropod's phenotype referenced (if more specific, then score this as yes and select appropriate trait(s) below)	*spider … looks creepy*
	legs		*six legs two small wings … insects rule the world*
	antenna		*Uncoil a copper antenna*
	mouthparts		*… long proboscis sucking blood … drawing nourishment"*
	head		*… she devours his head Deadly mantis love*
	wings		*… fluttering dance-like black-tipped wings …*
	genitalia	but not defensive structures	
	defensive structure	an anatomical structure used in defense, e.g., sting or stinger, scent glands	*Scorpions …<br>A stinger full of poison*
	setae	scales, hairs, bristles	*Sprinkling scales green —<br>The pale luna moth spoils*
	color / pattern	refers to color, color pattern, and/or reflectance	*she noticed the bugs were … speckled*<br><br>*this brown cockroach*
	extended phenotype	webs, nests, cocoons, galls, leaf mines, leaf rolls, etc.	*restless grass . . .<br>a spider's web*

physiology<br><br>n/12	PHYSIOLOGY	some unspecified aspect of the arthropod's physiology referenced (if more specific score this as yes and select appropriate trait(s)	*a cicada … chirps more slowly*<br><br>(colder weather affects movement)
	digestion	including excretion but not feeding	*… decorative pattern... fly shit*
	molting	including exuviae	*Molting is a must*<br>*The vehicle is renewed…*
	metamorphosis	references to metamorphosis, transformation	*… Ecdysone is important*<br>*Metamorphosis*
	vision	arthropod is seeing	*ants crawl on sunflower … for the view*
	chemosensory	arthropod is smelling (olfactory) or tasting (gustatory)	*The pheromones that link them together*
	hearing	arthropod is listening	*… a praying mantis listens to…*
	neural	arthropod is thinking or performing a task that requires higher functioning; or reference to general neural biology	*bees … choosing …*<br>*on which bloom to land*
	thermoregulation	arthropod is trying to regulate its temperature	*A cricket crawls … over the hearth-- a cold night*
	bioluminescence	explicit reference to how the arthropod is emitting light	*one feeble light shining...firefly*
	phototaxis	arthropod is responding to light, positively or negatively	*into the bonfire …*<br>*a tiger moth*
	sex	sex of the arthropod is explicitly referred to and is relevant	*but soon he's singing...*<br>*katydid*
life stage<br><br>n/4	life stage is explicitly mentioned or can be reasonable determined from the words used (i.e., haiku is complex enough to understand the life stage)		

	egg	including oothecae and egg sacs	*lay your <u>eggs</u> come August, Little Tiger Moth*
	immature	e.g., nymph, naiad, or larva	*oh, <u>caterpillar</u><br>snuggle in your silk blanket*
	pupa	for holometabolan insects	*…I'll find those tsetse <u>pupae</u> 'ere their folks find me*
	imago	adult	*<u>bumblebee</u><br><u>visiting</u> a lady*
behavior (not reproduction)<br><br>n/15	BEHAVIOR	some unspecified aspect of the arthropod's behavior - excluding locomotion - referenced (if more specific score this as yes and select appropriate trait(s)	*Spiders <u>weaving</u> gracefully*
	mimicry	arthropod looks like something else	*… <u>mimics host</u> as it nibbles…*
	predation	arthropod is a predator	*a <u>fly</u> sways slowly-<br><u>spiders web</u>*
	prey	arthropod is prey	*a <u>fly</u> sways slowly-<br><u>spiders web</u>*
	aposematism	arthropod advertises its dangerous nature	*… <u>Yellow and black</u> …<br><u>Watch out</u>, it can <u>sting</u>!*
	parasitism	arthropod is explicitly behaving as a parasite or reference to effects of parasitism	*oh the itch you'll get<br>when that <u>mosquito bites</u>*
	foraging	searching for food or other resources (e.g., for nest-building)	*I <u>follow the mosquito's whine</u> to the cheap inn*<br><br>*jittery hornet<br><u>chewing a chair</u>*
	pollination	arthropod pollinating flower	*Butterflies flutter… help <u>spread pollen</u>*
	feeding	any feeding that is neither predation nor parasitism	*insatiable minions, who <u>gorge</u>*
	defense	arthropod is defending	*<u>Stinging</u> the intruder*

		itself by stinging, biting, running away, etc.	
	sound production	arthropod is making sound	*The last cricket's chirps*
	sociality	refers to some element of the sociality spectrum: castes, gregariousness, intra-species interaction	*how lucky vicious fire ant queen*
	migration	arthropod is moving to a distant locality, as part of a seasonal change	*… monarch butterflies … on the way to home*
	dispersal	arthropod is moving for the purpose of finding food, a mate, or some other undefined reason	*all the baby spiders scatter*<br>*to make a living*
	grooming	arthropod grooming itself	*rubbing sand from his eyes … little butterfly*
ecology<br><br>n/8	ECOLOGY	some unspecified aspect of the arthropod's ecology referenced (if more specific score this as yes and select appropriate trait(s)	*blowing along...*<br>*a little butterfly*<br><br>(probably outside but unclear if natural or domestic)
	domestic	ecological context is human-related, including human body or in or near a house (peridomestic)	*roach now is hiding … in the seam underneath the white kitchen cabinets*
	peridomestic	Around a home or building, not inside	*Flew under the eaves*
	not domestic	clearly in a natural environment	*high desert*<br>*… surrounded*<br>*by a fly*
	association	inter-species interaction, excluding parasitism and predation	*Ants herd aphids …*
	paleontological	referring to fossils, deep evolutionary history	*trapped in amber*<br>*thousands of years ago*<br>*a honey bee*
	phenological	referencing the	*February sun*

		arthropod's seasonality	*under my thumb the first mosquito*
	temporal	reference includes information about whether arthropod is nocturnal, diurnal, etc.	*the horse's fart*<br>*wakes me to see...*<br>*fireflies flitting*

Entomological references

The following characteristics relate to arthropods as pests and to methods of pest management.

trait	description	examples from poems
pesticide	chemical control, including sprays	*the expert with his white tank of poison*<br><br>*a new cruelty, not just obvious roach spray*
cultural control	swatting, smashing, removing infestation manually	*pinching head lice …*<br><br>*my mosquito net's holes...*
biocontrol	refers to pests being controlled by another organism	*Bright red ladybugs*<br>*… Want to eat aphids.*
stored product pest	referring to arthropods infesting food stores (e.g., flour)	*In his cupboard*<br>*not a single moth*
nuisance	inconvenience pest - no serious harm, but is creepy, annoying, gross, etc.	*Despoilers all. Beetle, silverfish, cockroach*
structural pest	damages wood, including books	*From a worm-eaten cupboard*<br>*quiet drilling*<br><br>*Silverfish, tell me,*<br>*Darwin and Dostoevsky,*<br>*do they taste the same?*
agricultural pest	arthropod is a plant or garden pest, for example	*among the cabbage leafs*<br>*the white butterfly*
medical pest	not merely biting but also spreading disease (malaria, plague, typhus,	*Sucking up your blood*<br>*You may get malaria …*

	etc.)	
veterinary pest	arthropod is a pest of livestock or pets	*Under my dog's fur*<br>*A tick sucking blood at night …*
useful products	arthropod products mentioned, e.g., silk, honey, wax	*The Queen Bee is the Boss*<br>*they make honey*
collecting	mentions collecting, collections, preparing arthropods; refers to hobby or science	*cuticle impaled;*<br>*endless years of waiting*<br>*for one expert's glance*

Literary traits

The following characteristics relate to how the arthropod is referenced.

trait	description	examples from poems
outlandish	Is the biology believable or sensational / mythological?	*a butterfly flits–* (plausible)<br><br>*Earwig Jim … inside your head he gnaws away* (not plausible)
onomatopoeia	words that mimic the insects sounds are present	*buzz, buzz …*<br><br>*cicadas chirr …*
anthropomorphism	arthropod is ascribed human characteristics	*a butterfly deigns*<br>*to come and dance…*<br><br>*millipede mothers*<br>*… all those shoe laces*

Data cleaning

Open in OpenRefine (https://openrefine.org/; version 3.6.2 [579a6f7])

1. trim leading and trailing white spaces
2. change "yes" scores to 1 and "no" scores to 0; blanks stay blank
3. revisit blanks for each of the major categories (ANATOMY, PHYSIOLOGY, etc.) and score appropriately

4. use text facet function to look for taxon misspellings

Move dataset back into Google Sheets

5. calculate counts (sum) for each category (column) of scoring
6. look for outliers - e.g., categories with suspiciously low “yes” scores; entries in notes that ask questions or seek a second pair of eyes
7. sort by taxon and look for inconsistencies (e.g., should all Lampyridae have yes scores for bioluminescence? Are all poems with “webs” referring to extended phenotypes of Araneae?)
8. compare diagnostic words to taxon name; look for inaccuracies
9. sort by traits that proved confusing during scoring - e.g., phenology, temporal, genitalia, feeding
10. Sort by taxon and compare keywords to taxon; check taxon against NCBI taxonomy
11. Add formulas for scoring complexity (# traits scored yes / # total possible traits * 100)

---

useful ref for coding:

Given, L. M. (2008). Inter- and intracoder reliability. In The SAGE encyclopedia of qualitative research methods (pp. 446-446). SAGE Publications, Inc., https://dx.doi.org/10.4135/9781412963909.n223
