# Supplementary material for: Diversity and complexity of arthropod references in haiku
Source: PLoS One. 2024 Apr 3;19(4):e0298865. doi: 10.1371/journal.pone.0298865 (PMC10990216; doi:10.1371/journal.pone.0298865)
Supplement: S3 Appendix — This document describes the cluster analyses and results using the AntConc application. This document is also available through Penn State’s institutional repository, ScholarSphere: https://doi.org/10.26207/35sv-ep54. (PDF) [file pone.0298865.s010.pdf]

AntConc Cluster Analyses

Primary corpus

The primary haiku corpus was loaded into AntConc (version 4.1.3). After loading a list of stop words (appended below), a cluster analysis (word fragments; size=2; min freq=2; term position=left/right) was performed for each of the following arthropod-related terms. Hymenoptera, however, required an advanced search; see below.

1. spider

Cluster	Rank	Freq	Range	NormFreq	NormRange
spider s	3	28	1	0.141	1
spider web	4	12	1	0.06	1
spider webs	5	8	1	0.04	1
spider weaves	6	6	1	0.03	1
spider crawls	8	4	1	0.02	1
spider silk	8	4	1	0.02	1
jumping spider	8	4	1	0.02	1
spider moves	11	3	1	0.015	1
spider descends	12	2	1	0.01	1
spider thread	12	2	1	0.01	1
spider waits	12	2	1	0.01	1
spiders weaving	12	2	1	0.01	1
spiders web	12	2	1	0.01	1
baby spiders	12	2	1	0.01	1
black spider	12	2	1	0.01	1
hungry spider	12	2	1	0.01	1
little spider	12	2	1	0.01	1
small spider	12	2	1	0.01	1
tiny spider	12	2	1	0.01	1
water spider	12	2	1	0.01	1
wolf spider	12	2	1	0.01	1

2. dragonfl*|darner|damsel*|odon*|calopt*

Cluster	Rank	Freq	Range	NormFreq	NormRange
red dragonfly	3	16	1	0.094	1
dragonfly s	5	8	1	0.047	1
blue dragonfly	6	5	1	0.029	1
dragonfly hovers	9	4	1	0.023	1
dragonflies hover	11	3	1	0.018	1
dragonfly flying	11	3	1	0.018	1
dragonfly lands	11	3	1	0.018	1
eyes dragonfly	11	3	1	0.018	1
dragonfly dragonfly	19	2	1	0.012	1
dragonfly perched	19	2	1	0.012	1
dragonfly rests	19	2	1	0.012	1
dragonfly scowling	19	2	1	0.012	1
dragonfly shadows	19	2	1	0.012	1
dragonfly still	19	2	1	0.012	1
dragonfly wings	19	2	1	0.012	1
butterflies dragonflies	19	2	1	0.012	1
dragonfly dragonfly	19	2	1	0.012	1
one dragonfly	19	2	1	0.012	1

3. crick*|katy*|grassh*

Cluster	Rank	Freq	Range	NormFreq	NormRange
cricket s	6	13	1	0.042	1
crickets chirp	10	6	1	0.019	1
cricket chirps	12	5	1	0.016	1
cricket song	16	4	1	0.013	1
cricky cricky	16	4	1	0.013	1
grasshopper s	16	4	1	0.013	1
cricky cricky	16	4	1	0.013	1
one cricket	16	4	1	0.013	1
cricket sings	24	3	1	0.01	1
evening crickets	24	3	1	0.01	1
night cricket	24	3	1	0.01	1

night crickets	24	3	1	0.01	1
singing katydid	24	3	1	0.01	1
cricket band	34	2	1	0.006	1
cricket chirping	34	2	1	0.006	1
cricket crawls	34	2	1	0.006	1
cricket cricky	34	2	1	0.006	1
crickets chirping	34	2	1	0.006	1
crickets sing	34	2	1	0.006	1
crickets still	34	2	1	0.006	1
katydid chirrs	34	2	1	0.006	1
katydid little	34	2	1	0.006	1
katydid plum	34	2	1	0.006	1
katydid s	34	2	1	0.006	1
katydid sings	34	2	1	0.006	1
chirping cricket	34	2	1	0.006	1
cricket cricky	34	2	1	0.006	1
faint cricket	34	2	1	0.006	1
last cricket	34	2	1	0.006	1

4. aphid*|cicad*|stinkbug|bedbug|assassin|shield|fart|stink|strider

Cluster	Rank	Freq	Range	NormFreq	NormRange
cicada s	3	17	1	0.062	1
water strider	4	14	1	0.051	1
cicada chirrs	6	8	1	0.029	1
cicadas chirr	6	8	1	0.029	1
first cicada	6	8	1	0.029	1
cicada shell	10	6	1	0.022	1
cicadas sing	11	5	1	0.018	1
fart bug	11	5	1	0.018	1
water striders	11	5	1	0.018	1
cicada chorus	14	4	1	0.015	1
cicada sings	14	4	1	0.015	1
cicada cries	19	3	1	0.011	1

cicada cry	19	3	1	0.011	1
cicada husk	19	3	1	0.011	1
cicada singing	19	3	1	0.011	1
cicada song	19	3	1	0.011	1
cicadas singing	19	3	1	0.011	1
stink bug	19	3	1	0.011	1
singing cicada	19	3	1	0.011	1
summer cicada	19	3	1	0.011	1
summer cicadas	19	3	1	0.011	1
cicada still	36	2	1	0.007	1
stink bugs	36	2	1	0.007	1
autumn cicada	36	2	1	0.007	1
away cicada	36	2	1	0.007	1
away fart	36	2	1	0.007	1
first cicadas	36	2	1	0.007	1
last cicada	36	2	1	0.007	1
many cicadas	36	2	1	0.007	1
moon cicada	36	2	1	0.007	1
mountain cicada	36	2	1	0.007	1
night cicadas	36	2	1	0.007	1
one cicada	36	2	1	0.007	1
s fart	36	2	1	0.007	1
shrill cicadas	36	2	1	0.007	1

5. ant, ants, bee, bees, hornet, wasp, sawfly, sawflies (done as advanced search with this word list); irrelevant results in gray

Cluster	Rank	Freq	Range	NormFreq	NormRange
bee s	3	7	1	0.034	1
wasp nest	8	4	1	0.02	1
ants hell	11	3	1	0.015	1
ants scurry	11	3	1	0.015	1
ants hell	11	3	1	0.015	1
ants scurry	11	3	1	0.015	1
bee stings	11	3	1	0.015	1

beetle beetle	11	3	1	0.015	1
ant s	24	2	1	0.01	1
ant walks	24	2	1	0.01	1
ants autumn	24	2	1	0.01	1
ants come	24	2	1	0.01	1
ants crawling	24	2	1	0.01	1
ants march	24	2	1	0.01	1
distant thunder	24	2	1	0.01	1
mantis s	24	2	1	0.01	1
mantis sways	24	2	1	0.01	1
mantis waits	24	2	1	0.01	1
stagnant water	24	2	1	0.01	1
ants autumn	24	2	1	0.01	1
ants come	24	2	1	0.01	1
ants crawling	24	2	1	0.01	1
ants march	24	2	1	0.01	1
bee hums	24	2	1	0.01	1
beehive dangles	24	2	1	0.01	1
bees butterflies	24	2	1	0.01	1
bees buzz	24	2	1	0.01	1
bees buzzing	24	2	1	0.01	1
bees fly	24	2	1	0.01	1
beetle tries	24	2	1	0.01	1
bees butterflies	24	2	1	0.01	1
bees buzz	24	2	1	0.01	1
bees buzzing	24	2	1	0.01	1
bees fly	24	2	1	0.01	1
hornets nest	24	2	1	0.01	1
wasp circles	24	2	1	0.01	1
wasps nest	24	2	1	0.01	1

6. beet*|ladyb*|firef*|grub|lightningb*|coleop*

Cluster	Rank	Freq	Range	NormFreq	NormRange

first firefly	2	30	1	0.061	1
flitting firefly	4	16	1	0.033	1
calling fireflies	9	11	1	0.022	1
fireflies flit	14	7	1	0.014	1
firefly flits	17	6	1	0.012	1
firefly s	17	6	1	0.012	1
flitting fireflies	17	6	1	0.012	1
fireflies come	21	5	1	0.01	1
fireflies flitting	21	5	1	0.01	1
come firefly	21	5	1	0.01	1
firefly first	26	4	1	0.008	1
firefly flies	26	4	1	0.008	1
big firefly	26	4	1	0.008	1
dung beetle	26	4	1	0.008	1
beetle beetle	36	3	1	0.006	1
fireflies one	36	3	1	0.006	1
firefly don	36	3	1	0.006	1
firefly lights	36	3	1	0.006	1
beetle beetle	36	3	1	0.006	1
departing firefly	36	3	1	0.006	1
one firefly	36	3	1	0.006	1
beetle tries	48	2	1	0.004	1
fireflies firefly	48	2	1	0.004	1
fireflies fly	48	2	1	0.004	1
fireflies light	48	2	1	0.004	1
fireflies reed	48	2	1	0.004	1
fireflies though	48	2	1	0.004	1
fireflies visit	48	2	1	0.004	1
firefly can	48	2	1	0.004	1
firefly comes	48	2	1	0.004	1
firefly deftly	48	2	1	0.004	1
firefly firefly	48	2	1	0.004	1
firefly fly	48	2	1	0.004	1
firefly guest	48	2	1	0.004	1
firefly jar	48	2	1	0.004	1

firefly lands	48	2	1	0.004	1
firefly night	48	2	1	0.004	1
firefly still	48	2	1	0.004	1
firefly though	48	2	1	0.004	1
firefly uncaught	48	2	1	0.004	1
ladybug come	48	2	1	0.004	1
ladybug lady	48	2	1	0.004	1
ladybug rests	48	2	1	0.004	1
ladybugs crawling	48	2	1	0.004	1
away firefly	48	2	1	0.004	1
back fireflies	48	2	1	0.004	1
black beetle	48	2	1	0.004	1
chasing fireflies	48	2	1	0.004	1
fireflies firefly	48	2	1	0.004	1
firefly firefly	48	2	1	0.004	1
go firefly	48	2	1	0.004	1
grass fireflies	48	2	1	0.004	1
headstand beetle	48	2	1	0.004	1
huge firefly	48	2	1	0.004	1
japanese beetle	48	2	1	0.004	1
last firefly	48	2	1	0.004	1
myriad fireflies	48	2	1	0.004	1

7. mosquit*|maggot|midge|horsef*|crane

Cluster	Rank	Freq	Range	NormFreq	NormRange
mosquito net	3	29	1	0.074	1
mosquito larvae	4	16	1	0.041	1
mosquito s	5	12	1	0.03	1
mosquito swarm	5	12	1	0.03	1
s mosquitoes	7	11	1	0.028	1
pesky mosquito	11	7	1	0.018	1
mosquito smudge	13	6	1	0.015	1
mosquitoes whine	16	5	1	0.013	1

one mosquito	16	5	1	0.013	1
mosquito nets	20	4	1	0.01	1
swarming mosquitoes	20	4	1	0.01	1
whining mosquitoes	20	4	1	0.01	1
mosquito larva	26	3	1	0.008	1
mosquito spurting	26	3	1	0.008	1
mosquito whines	26	3	1	0.008	1
fleas mosquitoes	26	3	1	0.008	1
paper mosquito	26	3	1	0.008	1
temple mosquitoes	26	3	1	0.008	1
mosquito also	38	2	1	0.005	1
mosquito buzzes	38	2	1	0.005	1
mosquito eating	38	2	1	0.005	1
mosquito evening	38	2	1	0.005	1
mosquito lands	38	2	1	0.005	1
mosquito late	38	2	1	0.005	1
mosquito one	38	2	1	0.005	1
mosquito punk	38	2	1	0.005	1
mosquito swarms	38	2	1	0.005	1
mosquito thin	38	2	1	0.005	1
mosquitoes bite	38	2	1	0.005	1
mosquitoes buzz	38	2	1	0.005	1
mosquitoes buzzing	38	2	1	0.005	1
mosquitoes come	38	2	1	0.005	1
mosquitoes living	38	2	1	0.005	1
mosquitoes swarm	38	2	1	0.005	1
mosquitoes thin	38	2	1	0.005	1
mosquitoes two	38	2	1	0.005	1
afternoon mosquito	38	2	1	0.005	1
bedroom mosquito	38	2	1	0.005	1
burning mosquitoes	38	2	1	0.005	1
come mosquitoes	38	2	1	0.005	1
dancing mosquitoes	38	2	1	0.005	1
evening mosquitoes	38	2	1	0.005	1
first mosquito	38	2	1	0.005	1

gate mosquito	38	2	1	0.005	1
grass mosquitoes	38	2	1	0.005	1
hate mosquitoes	38	2	1	0.005	1
moon mosquito	38	2	1	0.005	1
swarm mosquitoes	38	2	1	0.005	1
thin mosquito	38	2	1	0.005	1
thin mosquitoes	38	2	1	0.005	1
today mosquito	38	2	1	0.005	1
well mosquitoes	38	2	1	0.005	1
whining mosquito	38	2	1	0.005	1

8. butterf*|moth|caterpil*|inchw*

Cluster	Rank	Freq	Range	NormFreq	NormRange
little butterfly	2	66	1	0.078	1
butterfly flits	5	16	1	0.019	1
butterfly s	7	14	1	0.016	1
tiger moth	9	12	1	0.014	1
white butterfly	9	12	1	0.014	1
blue butterfly	13	11	1	0.013	1
flitting butterfly	13	11	1	0.013	1
butterfly lands	19	8	1	0.009	1
monarch butterfly	19	8	1	0.009	1
white butterflies	19	8	1	0.009	1
first butterfly	26	7	1	0.008	1
meadow butterfly	26	7	1	0.008	1
spring butterfly	26	7	1	0.008	1
butterfly rests	33	5	1	0.006	1
meadow butterflies	33	5	1	0.006	1
spring butterflies	33	5	1	0.006	1
yellow butterfly	33	5	1	0.006	1
butterflies dance	42	4	1	0.005	1
butterflies fly	42	4	1	0.005	1
butterfly net	42	4	1	0.005	1

butterfly one	42	4	1	0.005	1
butterfly sleeps	42	4	1	0.005	1
moth s	42	4	1	0.005	1
black butterfly	42	4	1	0.005	1
little butterflies	42	4	1	0.005	1
luna moth	42	4	1	0.005	1
white moth	42	4	1	0.005	1
butterflies flitting	56	3	1	0.004	1
butterfly also	56	3	1	0.004	1
butterfly clinging	56	3	1	0.004	1
butterfly come	56	3	1	0.004	1
butterfly dances	56	3	1	0.004	1
butterfly flying	56	3	1	0.004	1
butterfly little	56	3	1	0.004	1
butterfly wings	56	3	1	0.004	1
moth flutters	56	3	1	0.004	1
mother s	56	3	1	0.004	1
autumn butterfly	56	3	1	0.004	1
blue butterflies	56	3	1	0.004	1
first butterflies	56	3	1	0.004	1
huge butterfly	56	3	1	0.004	1
one butterfly	56	3	1	0.004	1
two butterflies	56	3	1	0.004	1
butterflies dancing	78	2	1	0.002	1
butterflies dragonflies	78	2	1	0.002	1
butterflies gliding	78	2	1	0.002	1
butterflies kiss	78	2	1	0.002	1
butterfly alights	78	2	1	0.002	1
butterfly butterfly	78	2	1	0.002	1
butterfly came	78	2	1	0.002	1
butterfly dancing	78	2	1	0.002	1
butterfly dips	78	2	1	0.002	1
butterfly don	78	2	1	0.002	1
butterfly drifts	78	2	1	0.002	1
butterfly emerges	78	2	1	0.002	1

butterfly even	78	2	1	0.002	1
butterfly every	78	2	1	0.002	1
butterfly flitters	78	2	1	0.002	1
butterfly flitting	78	2	1	0.002	1
butterfly flutters	78	2	1	0.002	1
butterfly fly	78	2	1	0.002	1
butterfly hey	78	2	1	0.002	1
butterfly leaves	78	2	1	0.002	1
butterfly living	78	2	1	0.002	1
butterfly quivering	78	2	1	0.002	1
butterfly shadow	78	2	1	0.002	1
butterfly summer	78	2	1	0.002	1
butterfly take	78	2	1	0.002	1
butterfly visits	78	2	1	0.002	1
caterpillar big	78	2	1	0.002	1
caterpillar drops	78	2	1	0.002	1
inchworm dancing	78	2	1	0.002	1
moth clings	78	2	1	0.002	1
moth flies	78	2	1	0.002	1
moth lands	78	2	1	0.002	1
moth still	78	2	1	0.002	1
mother cat	78	2	1	0.002	1
moths circle	78	2	1	0.002	1
bees butterflies	78	2	1	0.002	1
big caterpillar	78	2	1	0.002	1
blossoms butterfly	78	2	1	0.002	1
brown moth	78	2	1	0.002	1
butterfly butterfly	78	2	1	0.002	1
cabbage moth	78	2	1	0.002	1
dancing butterflies	78	2	1	0.002	1
dead moths	78	2	1	0.002	1
flexible inchworm	78	2	1	0.002	1
flitting butterflies	78	2	1	0.002	1
fly butterfly	78	2	1	0.002	1
garden butterflies	78	2	1	0.002	1

green caterpillar	78	2	1	0.002	1
gypsy moth	78	2	1	0.002	1
haughty butterfly	78	2	1	0.002	1
high butterfly	78	2	1	0.002	1
monarch butterflies	78	2	1	0.002	1
new butterfly	78	2	1	0.002	1
one moth	78	2	1	0.002	1
rare butterfly	78	2	1	0.002	1
sleeping moth	78	2	1	0.002	1
sparrows butterflies	78	2	1	0.002	1
sphinx moth	78	2	1	0.002	1
still butterfly	78	2	1	0.002	1
stop butterfly	78	2	1	0.002	1
summer butterfly	78	2	1	0.002	1
tent caterpillar	78	2	1	0.002	1
yellow butterflies	78	2	1	0.002	1
yellow caterpillar	78	2	1	0.002	1

Stop words list

a
about
above
after
again
against
all
am
an
and
any
are
as
at
be
because
been
before
being
below
between
both
but
by
cannot
could
did
do
does
doing
down
during
each
few
for
from
further
had
has
have
having
he
her
here
hers
herself
him
himself
his
how
i
if
in
into

is
it
its
itself
me
more
most
my
myself
no
nor
not
of
off
on
once
only
or
other
ought
our
ours
out
over
own
same
she
should
so
some
such
than
that
the
their
theirs
them
themselves
then
there
these
they
this
those
through
to
too
under
until
up
very
was
we
were
what
when
where
which
while
who
whom
why
with
would
you
your
yours
yourself
yourselves

References

- Laurence Anthony. AntConc tutorial videos. https://www.youtube.com/user/AntlabJPN/
- Heather Froehlich, "Corpus Analysis with Antconc," Programming Historian 4 (2015), https://doi.org/10.46430/phen0043
